# Tracking DNA damage localization and chromatin remodeling in live cells using time-resolved quantitative analysis of DNA counterstains

**DOI:** 10.1007/s00018-026-06100-9

**Published:** 2026-02-16

**Authors:** Greta Paternò, Elisa Longo, Luca Lanzanò

**Affiliations:** 1 Department of Physics and Astronomy “Ettore Majorana”, University of Catania, Via S. Sofia 64, Catania, 95123 Italy; 2 Nanoscopy, CHT Erzelli, Istituto Italiano di Tecnologia, Via Enrico Melen 83, Building B, Genoa, 16152 Italy; 3 Istituto Nazionale di Fisica Nucleare (INFN) - Sezione di Catania, Via S. Sofia 64, Catania, 95123 Italy; 4 Centro Siciliano di Fisica Nucleare e Struttura della Materia (CSFNSM), Via S. Sofia 64, Catania, 95123 Italy

**Keywords:** DNA damage, Chromatin relaxation, DNA counterstain, Euchromatin, Heterochromatin, Image cross-correlation spectroscopy (ICCS), DNA density

## Abstract

DNA damage profoundly impacts genome stability and cellular homeostasis, and its repair is tightly coordinated with local chromatin remodeling. However, monitoring these rapid chromatin changes in living cells remains challenging. We recently developed QUANDO, an imaging-based method that exploits a simple DNA counterstain to investigate the subnuclear localization of DNA damage in fixed cells. Here, we adapt this approach to track chromatin remodeling at DNA damage sites in live cells using Hoechst-based staining. Specifically, PARP1-expressing HeLa cells are exposed to UV laser micro-irradiation in a defined nuclear region, and chromatin dynamics are monitored in real time. As expected, PARP1 rapidly accumulates at damage sites, but this response is markedly suppressed upon treatment with Talazoparib, a potent PARP inhibitor. We further observe that laser-induced DNA damage initially localizes to high-density chromatin regions, followed by rapid chromatin relaxation, as indicated by decreasing Hoechst intensity and coefficient of variation (CV). This relaxation is significantly impaired by Talazoparib, consistent with PARP1-dependent chromatin remodeling. Finally, Hoechst-only imaging provides comparable results in both transfected and non-transfected cells, underscoring the robustness of the method and its compatibility with single-label/single-channel acquisition. Chromatin relaxation kinetics were similar in transfected and non-transfected cells, confirming that staining with Hoechst is sufficient for studying chromatin dynamics bypassing the complexities of transfection. These findings underscore the dynamic interplay between DNA damage and chromatin remodeling, demonstrating how conventional nuclear counterstaining can reveal rapid chromatin changes at damage sites, offering new perspectives for investigating genome stability in live cells.

**Supplementary Information:**

The online version contains supplementary material available at 10.1007/s00018-026-06100-9.

## Introduction

The integrity of the genome is continuously exposed to endogenous and exogenous sources of DNA damage, including reactive oxygen species, replication stress, and environmental agents, which can result in single- and/or double-DNA strand breaks (SSBs and DSBs) [1, 7]. To preserve genomic stability, cells have evolved the DNA Damage Response (DDR), a complex signaling network that detects DNA damage, orchestrates repair pathways, and modulates cell fate decisions. In this process, chromatin remodeling plays a crucial role by facilitating access of repair factors to damaged sites and ensuring efficient processing of lesions [11, 24].

Among the earliest responses to DNA damage is the activation of poly(ADP-ribose) polymerase 1 (PARP1), which binds DNA breaks and catalyzes the formation of poly(ADP-ribose) chains (PAR) on itself and chromatin-associated proteins such as histones [15, 31, 36]. PAR signaling critically regulates chromatin architecture: while PARP1 binding may promote local over-compaction, its enzymatic activity induces chromatin relaxation, thereby enhancing DNA accessibility [34, 38]. This dynamic remodeling facilitates the timely recruitment of DNA-binding and repair factors by transiently reshaping the chromatin landscape at sites of damage, ensuring efficient coordination of the repair process [18, 36]. Given its pivotal role in DNA repair and chromatin regulation, PARP1 has become a key therapeutic target in tumors with homologous recombination deficiencies, such as BRCA1/2-mutated cancers [32], where PARP inhibitors (e.g., Olaparib, Niraparib, Rucaparib, Talazoparib) exert clinical efficacy by trapping PARP on DNA, blocking repair, and promoting the accumulation of cytotoxic lesions [10, 13, 17, 29].

Over the past decades, several microscopy-based studies have shown that chromatin is a highly dynamic structure undergoing extensive remodeling upon DNA damage, which critically influences repair efficiency and pathway choice [19, 22]. To investigate these remodeling events, particularly chromatin change of compaction at damage sites, various imaging strategies have been employed. Among them, a widely used approach involves laser micro-irradiation in combination with a sensitizer to induce localized DNA damage [37]. Laser micro-irradiation can be synchronized with time-lapse imaging of live cells to study early events in the DNA damage response. In particular, chromatin remodeling has been observed by monitoring the intensity of photoactivatable histones like H2B-PAGFP (which are photoactivated in the same region of laser-micro-irradiation). In this type of approach, fluorescently labeled chromatin domains expand within seconds to minutes after activation, revealing rapid local relaxation at DNA damage sites [3, 18, 34].

Here, we propose a simpler approach to quantitatively monitor chromatin remodeling using labeling with a vital dye. We demonstrate that DNA damage-induced chromatin reorganization can be effectively studied in live cells using an optimized workflow that combines confocal imaging with a conventional nuclear counterstain. In our approach, we use the DNA counterstain Hoechst 33342 both as a sensitizer and a chromatin remodeling marker. The data are analyzed using a method called QUantitative ANalysis of DNA cOunterstains (QUANDO) [27] that enables the measurement of DNA density level at damage sites. This method combines Image Cross-Correlation Spectroscopy (ICCS) [5, 6, 8, 26] which quantifies the spatial co-distribution between DNA damage markers and chromatin stains, with a DNA density analysis that estimates chromatin compaction levels based on counterstain intensity. Together, ICCS and DNA density offer complementary insights into the local chromatin environment, helping to distinguish between heterochromatin and euchromatin at sites of genomic damage [5, 6, 26, 27]. We applied this approach to HeLa cells transfected with a PARP1 chromobody and subjected to UV laser micro-irradiation using Hoechst as a sensitizing dye. First of all, we monitored the dynamics of PARP1 recruitment to ensure that DNA damage was effectively induced: as expected, we observed PARP1 accumulation at damage sites and that this recruitment dynamics was markedly slowed down following treatment with Talazoparib, a potent PARP1 inhibitor. Next, we found that laser-induced DNA damage initially accumulates in high DNA density regions, as indicated by the high values of DNA density and co-localization with heterochromatin that decrease over time. DNA damage is followed by rapid chromatin relaxation, as indicated by a decrease in Hoechst intensity and coefficient of variation (CV). The chromatin relaxation effect is impaired by Talazoparib treatment, which interferes with PARP1-dependent chromatin remodeling. Finally, we found that Hoechst-only imaging provided comparable results in both PARP1-transfected and non-transfected cells, highlighting the method’s robustness and its suitability for single-channel acquisition without requiring transfection.

## Materials and methods

### Cell culture, treatments and labeling

HeLa cells (ATCC n. CCL-2™) are cultured in DMEM (Dulbecco’s modified Eagle’s medium, Gibco™, 11965092) supplemented with 10% Fetal Bovine Serum (Sigma-Aldrich, F9665) and 1% Penicillin/Streptomycin (Sigma-Aldrich, P4333) and maintained at 37 °C and 5% CO_2_.

For fluorescence microscopy experiments, 30,000 cells/cm^2^ are seeded on 8-well Ibidi chambered coverslips and incubated at 37 °C and 5% CO_2_ for 24 h to reach approximately 80–90% confluence. Cells are then transiently transfected using Lipofectamine 3000^®^ transfection kit (Thermo Fisher Scientific, L3000-001) according to the manufacturer’s instructions, with a plasmid encoding a PARP1 chromobody tagged with TagRFP (PARP1-RFP) (ChromoTek, xcr).

After transfection, cells are further incubated for 24 h under the same conditions for subsequent treatments and live-cell imaging. Nuclear staining is performed by incubating the cells with 2 µM Hoechst 33342 (Thermo Fisher Scientific, 62249) for 15 min at 37 °C, followed by replacement with fresh medium. To inhibit PARP1 activity, cells are treated with 500 nM, 5 nM and 0.5 nM Talazoparib (Selleck Chemicals, BMN 673) for 45 min prior to imaging.

## Image acquisition

All measurements are performed on a Leica TCS SP8 confocal laser scanning microscope, using a 63×/1.40 NA oil-immersion objective (HCX PL APO CS2 63/1.40 Oil, Leica Microsystems). For live-cell imaging, the microscope stage was maintained at 37 °C with 5% CO₂ using a temperature- and CO₂-controlled incubation chamber.

Imaging is carried out using a 512 × 512 pixels format, line frequency of 8000 Hz (resonant scanner mode), zoom factor of 5.00, a pinhole size set to 1.00 Airy Unit with 10× line accumulation and a pixel size of 72 nm.

Excitation/emission wavelengths are the following: Channel 1 (Ch1) - PARP1-RFP (561/570–650) via hybrid detector operating in photon counting mode (power setting 0.5%); Channel 2 (Ch2) - Hoechst (405/415–510), via hybrid detector operating in photon counting mode (power setting 0.5%).

For the induction of DNA damage, laser micro-irradiation is performed using the 405 nm laser line at 50% power. A rectangular region of interest (ROI) of 18 μm × 4 μm is manually placed at the edge of the nucleus prior to bleaching. For Supplementary Fig. S1, a 6 μm × 4 μm ROI is manually positioned at the inner edge of the nucleus prior to bleaching. The time lapse imaging and bleach setting are the following:

- Pre-bleach: 5 iterations, time per iteration = 1.3 s.
- Bleach: 2 iterations, time per iteration = 1.3 s (total exposure time 2.6 s).
- Post-bleach: 50 iterations, time per iteration = 3 s.

In the time-lapse analysis, we define the beginning of the micro-irradiation as the time t = 0 so that t = 2.6 s corresponds to the first image acquired post-irradiation.

## Data processing

The acquired images are pre-processed on Fiji [33] to obtain the suitable input files for DNA density analysis and Image Cross-Correlation Spectroscopy (ICCS), with the QUANDO algorithm implemented in MATLAB (https://github.com/llanzano/QUANDO), as thoroughly described in Paternò et al. [27]. The input files are the following:

- “Nuclei selection count masks”: label images identifying each nucleus as a distinct object over time in the time-lapse sequence, used to define nuclear boundaries. They are generated as follows: the images of the DNA channel (Ch2) from the time-lapse sequence of the same nucleus are converted into binary images using the function ‘‘Threshold’’ of ImageJ, using the “Default” threshold algorithm. The nuclei are identified and listed as objects using the ‘‘Analyze particles’’ function and the images of the ‘‘Count Masks’’ are saved;
- “DNA damage area count masks”: label images representing the intersection between nuclei and the micro-irradiated ROI, representing the irradiated nuclear areas;
- “Binary masks of DNA damage foci”: binary images highlighting pixels corresponding to occurrence of DNA damage. The binary masks of DNA damage are generated by excluding background pixels from the PARP1 channel (Ch1), setting a fixed intensity threshold of 80% of the maximum intensity value;
- “Intensity images”: background-subtracted fluorescence images, used for quantitative intensity measurements. The intensity images are generated by smoothing the images and subtracting the background from the intensity images of nuclei channel (Ch2) using the function “Subtraction of Background” (rolling ball radius of 100 pixels). The rolling ball radius size corresponds approximately to the maximum size of heterochromatin features that we observed in our images.

## Image Cross-Correlation spectroscopy (ICCS) analysis

The Image Cross-Correlation Spectroscopy (ICCS) analysis is based on a modified version of the ICCS algorithm [26] (https://github.com/llanzano/ICCS), well described in [6, 30]. The algorithm is performed in MATLAB (The MathWorks, Natick, Massachusetts). We adapt the algorithm to perform automatic calculation of ICCS parameters on each time point of the time-lapse. For each time point, the main algorithm output is the parameter f_1_ (f_2_) values which represent the fraction of signal in channel 1 (channel 2) which is cross-correlated with the other channel. Values of this parameter range from 1 (maximum cross-correlation), to 0 (no cross-correlation), to − 1 (maximum anti-correlation) [6].

The colocalization fraction extracted by ICCS is analogous but not identical to the Pearson Correlation Coefficient [6]. The main difference is that the ICCS parameter is calculated using the image cross- and auto-correlation functions. Specifically, the cross-correlation fraction f_1_ is defined as the ratio between the amplitude (estimated at zero spatial lag) of the cross-correlation function and the amplitude (extrapolated at zero spatial lag) of auto-correlation function of channel 2.

In this application, we cross-correlate the signal corresponding to DNA damage (PARP1 channel) with the signal corresponding to the DNA marker (Hoechst channel). Thus, the value of the parameter f_1_ represents the fraction of colocalization of DNA damage with regions of high DNA density (heterochromatic regions). The analysis is limited to the irradiated area of the nucleus using “DNA damage area count masks” as the region of analysis.

## DNA density analysis

For each time point, we calculate the image of the normalized DNA intensity as:

1 $$\text I_\mathrm{DNA-norm}\left(\text {x, y}\right)=\text I_\mathrm{DNA}\left(\text {x, y}\right)/\text I_\text {max}$$

Where IDNA(x,y) is the intensity image of the DNA channel and Imax is the maximum value of IDNA(x,y) in the “Nuclei selection count masks”. 

Then we calculate the average value of the normalized DNA intensity in the region of interest (ROI):

2 $$\mathrm{DNA}\;\mathrm{density}=<{\mathrm I}_{\mathrm{DNA}-\mathrm{norm}}(\mathrm x,\mathrm y)>_\mathrm{ROI}$$

Where the ROI is represented by the intersection between the “Nuclei selection count masks” and the region defined by “Binary masks of DNA damage foci”. For each time-point, the value of “DNA density” represents the average value of the normalized DNA intensity, calculated at the DNA damage foci. We expect high values of DNA density if DNA damage overlaps with heterochromatic regions.

## Intensity and CV analysis

For each time-point, average values of PARP1 intensity (Ch1) and Hoechst intensity (Ch2) are calculated in the ROI defined by the “DNA damage area count masks”. The coefficient of variation (CV) of the intensity in the DNA channel is calculated as [25] :

3 $$\mathrm{CV}=\mathrm\sigma/\mathrm\mu$$

Where σ and µ are the standard deviation and the mean value of Hoechst intensity in the same ROI, respectively.

## Graphs and statistical analysis

Graphs and statistical analyses are performed using GraphPad Prism version 8.0.0 for Windows, GraphPad Software, San Diego, California USA, www.graphpad.com. For all the analyses, differences among two groups are analyzed by T-test. The values are expressed as mean ± s.d. in Fig. 2, mean ± s.e.m. in Figs. 3 and 4, 5, and a *p* < 0.05 is accepted as significant.

For the intensity time trace analysis, the accumulation of PARP1-RFP (T_on_) at damage sites is examined by fitting the short-term intensity profiles (average intensity within the DNA damage region) using the exponential one-phase association model in GraphPad Prism. The intensity profile during the decay phase (T_off_) is analyzed separately using the exponential one-phase decay equation.

## Results

### Hoechst-based approach to investigate chromatin dynamics following DNA damage

**Fig. 1:**
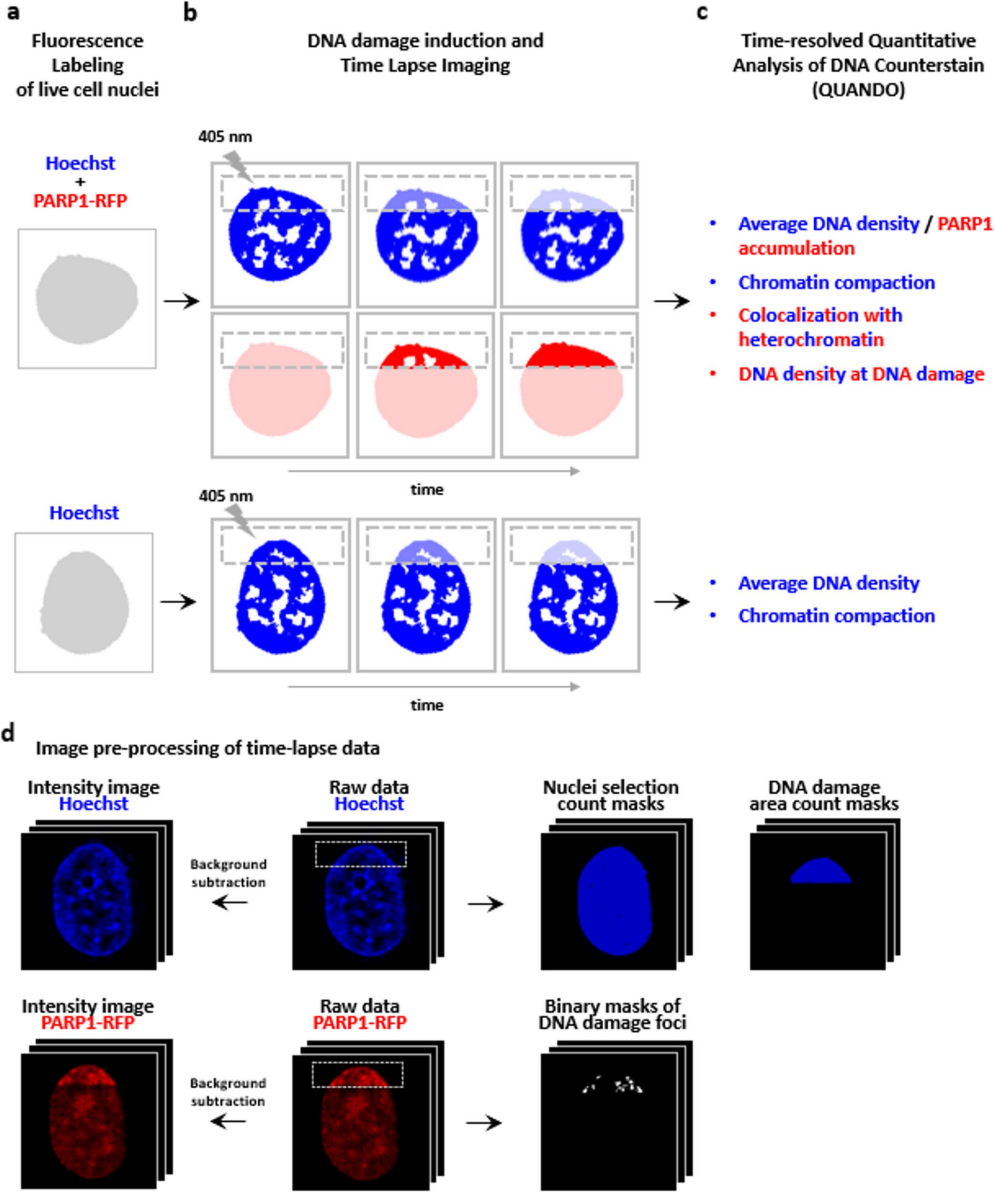
Schematic overview of the experimental workflow used to monitor chromatin remodeling at DNA damage sites in live cells. (**a**) Live cells were stained with Hoechst (blue) and included both PARP1-transfected (red) and non-transfected cells. (**b**) DNA damage was induced with 405 nm laser micro-irradiation in a specific nuclear region (dashed box), and time-lapse imaging was used to monitor PARP1 recruitment and chromatin dynamics. (**c**) Acquired images were processed with QUANDO to quantify DNA damage localization (ICCS and DNA density analysis) and chromatin remodeling (Hoechst intensity and CV). (**d**) Schematic description of the processing of time-lapse raw data and preparation of the images required to perform the analysis

To investigate chromatin remodeling at DNA damage sites in living cells, we established an experimental workflow combining Hoechst nuclear staining, laser micro-irradiation, time-lapse imaging and a quantitative image analysis (Fig. 1). Notably, Hoechst also acts as a photosensitizer: upon UV excitation, it generates reactive oxygen species, enabling the induction of site-specific DNA damage in live cells [14]. As illustrated schematically in Fig. 1a, the method can be applied to PARP1-transfected and non-transfected cells. In both cases, nuclei were labeled with Hoechst, and localized DNA damage was induced by a focused 405 nm laser within a defined nuclear region (dashed box). Time-lapse imaging allowed simultaneous monitoring of PARP1 recruitment to damage sites (only in PARP1-transfected cells) and chromatin remodeling over time (Fig. 1b). Regions of interest (ROI) were defined around the irradiated area to track the evolution of fluorescence signals.

Following image acquisition, we processed the data using a time-resolved QUANDO analysis. In PARP1-transfected cells we evaluated DNA damage localization by: (i) measuring the colocalization of DNA damage with heterochromatin regions using Image Cross-Correlation Spectroscopy (ICCS), and (ii) analyzing changes in local chromatin density through normalized Hoechst intensity at the damaged area as shown in [27]. We expect higher colocalization and DNA density values when DNA damage occurs in heterochromatic regions, and lower values when damage localizes to euchromatin. In parallel, we quantified the average PARP1 signal intensity as a readout of PARP1 recruitment, providing insights into the protein’s dynamic response to DNA damage, including under conditions where PARP activity is inhibited by Talazoparib (see below). In both transfected and non-transfected cells, we evaluated chromatin remodeling, using the Hoechst signal intensity and its coefficient of variation (CV). The CV reflects the heterogeneity of nuclear staining: higher Hoechst intensity and CV values are typically associated with more compact chromatin, while lower values indicate chromatin relaxation (Fig. 1c). The whole time-resolved analysis is automated thanks to the generation of count masks that identify the region of analysis for each time point (Fig. 1d).

### PARP1 accumulation following laser-induced DNA damage

**Fig. 2:**
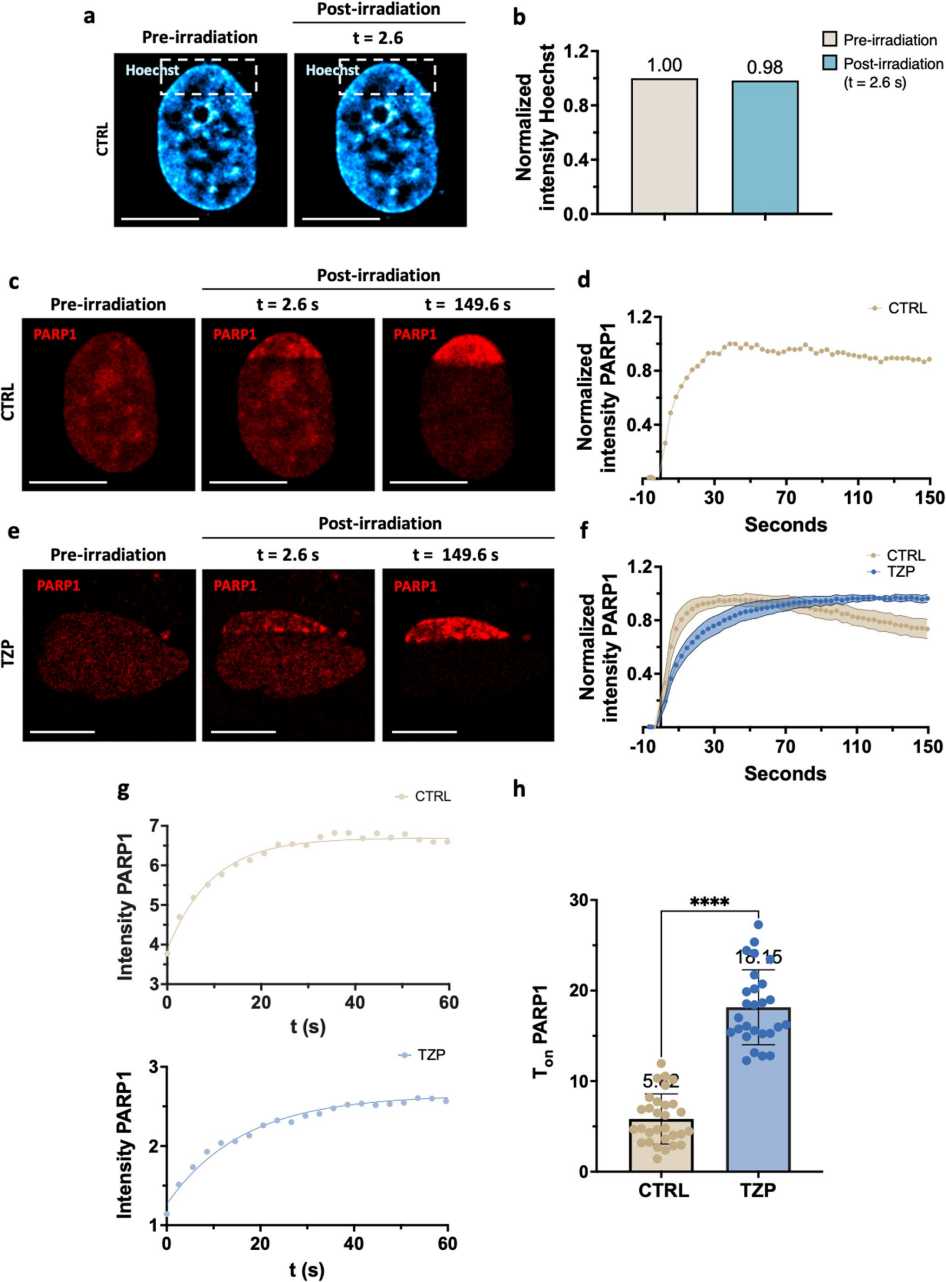
Time-lapse imaging of PARP1 dynamics following DNA damage. (**a**) Representative images of live HeLa cell nuclei stained with Hoechst in cyan-hot LUT before and immediately after irradiation (t = 2.6 s). Dashed box represents the defined nuclear region targeted by laser-induced DNA damage. Scale bars: 10 μm. (**b**) Comparison of normalized intensity Hoechst between pre-irradiation and post-irradiation. T-Test was applied and no significant difference was detected. (**c–e**) Confocal images show PARP1 recruitment at the damage site in control (CTRL) and Talazoparib-treated cells (TZP). For each condition, three time points are shown: pre-irradiation, and post-irradiation at t = 2.6 s and t = 149.6 s. Scale bar: 10 μm. (**d–f**) Normalized intensity profiles of PARP1 over time in control and Talazoparib-treated cells. (**f**) Cloud plots represent mean ± s.d. from 3 independent experiments, with approximately 10 cells analyzed per experiment, for a total of ~ 30 cells. (**g**) Representative fitting curves using a one-phase association model (Tₒₙ) of PARP1 accumulation in control and Talazoparib-treated cells within the first 60 s. h) Dot plots showing the comparison of PARP1 Tₒₙ values between control and Talazoparib-treated cells. Data points represent mean ± s.d. from 3 independent experiments, with approximately 10 cells analyzed per experiment, for a total of ~ 30 cells, T-Test ****p < 0.0001

In order to use Hoechst both as a sensitizer and a chromatin marker, we set up a configuration in which the laser micro-irradiation procedure did not cause significant photobleaching of the Hoechst dye. To this end, we compared Hoechst fluorescence intensity immediately before and 2.6 s after micro-irradiation. No significant changes were detected, as confirmed qualitatively by confocal images (Fig. 2a) and quantitatively by normalized intensity plots (Fig. 2b). Nevertheless, laser irradiation effectively induced DNA damage, as demonstrated by PARP1 accumulation at the irradiated region of interest (ROI) (Fig. 2c). To capture a broad chromatin landscape and better visualize spatial organization, the ROI was designed with a rectangular shape (approx. 72 μm²), encompassing an extended nuclear area. Before the micro-irradiation (pre-irradiation), confocal imaging revealed that in control cells (Fig. 2c), PARP1 was uniformly distributed throughout the nucleoplasm, with a higher concentration observed in the nucleoli, in keeping with previous reports [2, 20]. Immediately after damage induction (2.6 s post-irradiation), PARP1 rapidly accumulated at the targeted site, forming distinct high-intensity foci. By the end of the time-lapse acquisition (149.6 s post-irradiation), the fluorescence signal appeared more diffuse and evenly distributed across the ROI. In contrast, in cells pre-treated with the PARP inhibitor Talazoparib (Fig. 2e), PARP1 dynamic was altered. Although the initial accumulation at the damage site resembled that of the control cells, by the end of the time-lapse acquisition the fluorescence signal remained confined to well-defined foci, preserving a structured pattern. This behavior suggests a delayed yet more stable binding of PARP1 in the presence of the inhibitor. Importantly, we observed that this behavior was conserved also when DNA damage was induced in an internal nuclear region. Specifically, when the ROI was positioned inside the nucleus (area of approx. 24 μm²), both control and Talazoparib-treated cells displayed PARP1 recruitment dynamics consistent with those observed at the nuclear periphery, as shown in Supplementary Fig. S1a-b.

To characterize these dynamics, fluorescence intensity values of PARP1 were calculated over time. In Fig. 2d, we reported the normalized intensity profile of PARP1 in a single untreated control cell. Figure 2f showed the averaged intensity profiles of untreated (beige) and Talazoparib-treated (light blue) cells. In control cells, PARP1 intensity rapidly increased, reached its peak at 53.6 s (the normalization reference point) and then gradually declined. In contrast, Talazoparib-treated cells displayed a delayed peak at 134.6 s, with a slower accumulation rate and no subsequent decrease, resulting in a sustained plateau. These observations indicate that PARP1 binding was delayed but exhibited increased stability in the presence of the inhibitor.

To quantitatively assess the recruitment kinetics, we fitted the intensity curves using a single-exponential function (Fig. 2g), which estimates the characteristic time required for PARP1 accumulation at DNA damage sites. The recruitment time constant (Tₒₙ) was significantly higher in Talazoparib-treated cells (light blue), with Tₒₙ = 18.15 s ± 4.1 (mean ± s.d. from 30 cells), compared to untreated controls (beige), which showed a Tₒₙ = 5.82 s ± 2.8 (mean ± s.d. from 30 cells), as reported in Fig. 2h. The slower yet sustained accumulation is consistent with the PARP trapping effect, in which Talazoparib impairs PARP1 dissociation from DNA damage sites and promotes its prolonged retention on chromatin, thereby slowing down the accumulation kinetics. Importantly, similar results were observed when DNA damage was induced in an internal nuclear region. As shown in Supplementary Fig. S1c-d, both control and Talazoparib-treated cells displayed normalized PARP1 intensity profiles and corresponding Tₒₙ values consistent with those observed at the nuclear periphery, with mean Tₒₙ = 2.93 s ± 1.4 (mean ± s.d. from 8 cells) for controls and Tₒₙ = 16.68 s ± 1.4 (mean ± s.d. from 8 cells) for Talazoparib-treated cells. These data indicate that PARP1 recruitment kinetics are quantitatively analogous in internal and peripheral nuclear regions.

### Time-resolved QUANDO-based quantification of PARP1 localization after laser-induced DNA damage

Next, we applied the QUANDO method to analyze the localization of laser-induced DNA damage in control and Talazoparib-treated cells. Specifically, we tracked the recruitment of endogenous PARP1 over time following 405 nm laser micro-irradiation. PARP1 localization was evaluated using the parameters: (i) DNA density, and (ii) colocalization with heterochromatin (see Methods). As previously demonstrated by [27], high DNA density values combined with strong colocalization with heterochromatin indicate that DNA damage preferentially localizes within more compact chromatin regions. Conversely, low DNA density and reduced colocalization suggest a localization of the damage signal into euchromatic regions.

**Fig. 3:**
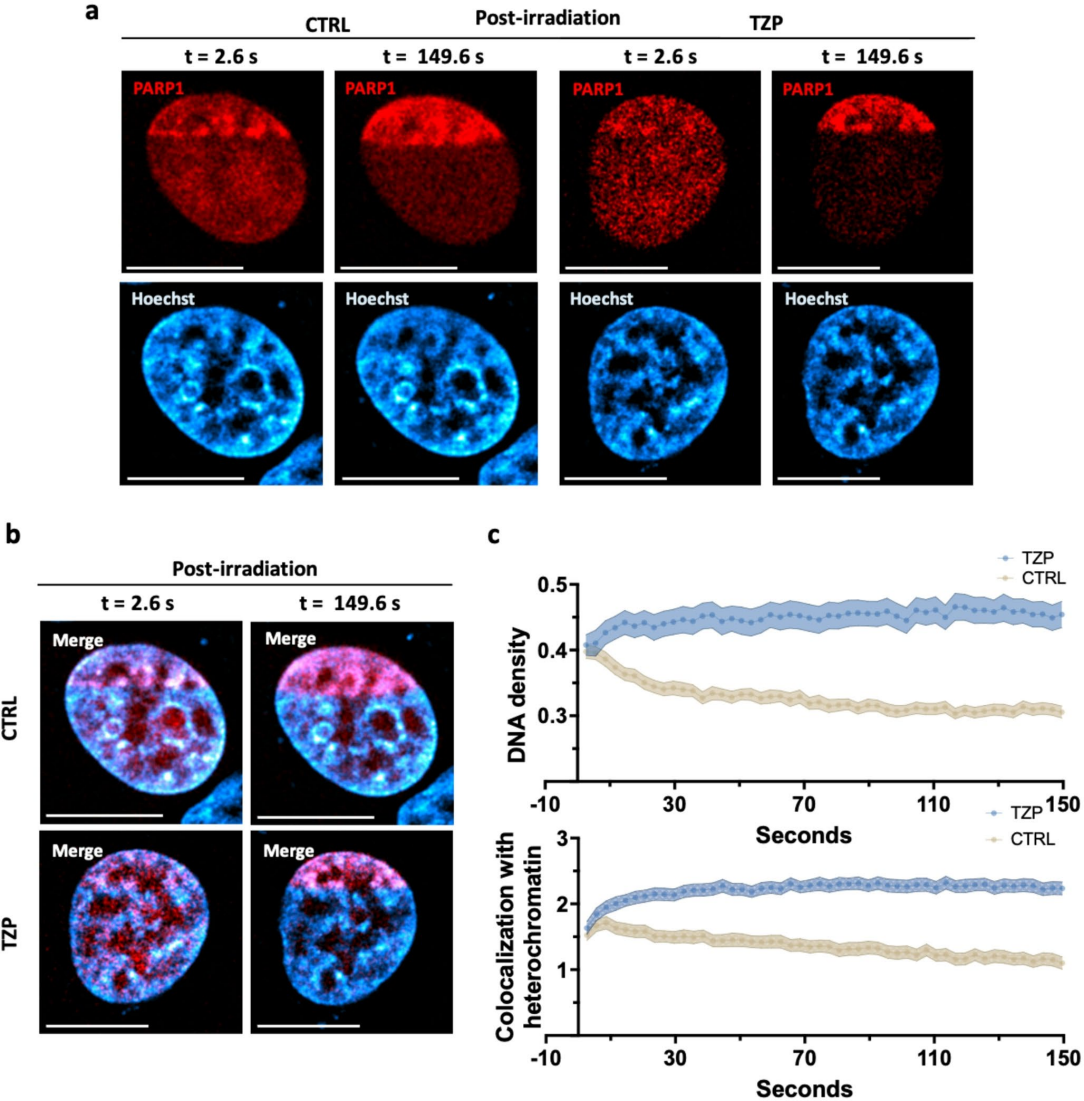
PARP1 localization dynamics revealed by time-resolved QUANDO. (**a-b**) Representative confocal images of control (CTRL) and Talazoparib-treated (TZP) HeLa cells at early (t = 2.6 s) and late (t = 149.6 s) time points after laser-induced DNA damage. Images show PARP1 (red) and DNA counterstaining with Hoechst (cyan-hot LUT) and the corresponding merged channels. (**c**) Top: DNA density corresponding to PARP1 accumulation over time in control (beige) and Talazoparib-treated (blue) cells. Cloud plots show the mean ± s.e.m. of 3 independent experiments, with approximately 10 cells analyzed per experiment, for a total of ~ 30 cells. Bottom: Colocalization of PARP1 with heterochromatin over time in control (beige) and Talazoparib-treated (blue) cells. Pattern cloud plots show the mean ± s.e.m. of 3 independent experiments, with approximately 10 cells analyzed per experiment, for a total of ~ 30 cells

As shown in Fig. 3a-b, confocal images acquired immediately after laser micro-irradiation (t = 2.6 s) revealed that PARP1 (red) rapidly accumulated at the damage site and preferentially localized to regions of high Hoechst intensity (cyan-hot LUT), which corresponded to more compact chromatin. Then, control cells displayed a progressive diffusion of the PARP1 signal, which became less confined and more evenly distributed across the damaged area, suggesting that, as the DNA damage response progressed, PARP1 spread into lower-density, euchromatic regions. In contrast, Talazoparib-treated cells maintained a more restricted and structured PARP1 distribution, indicating sustained accumulation within dense chromatin compartments.

Within the first ~ 10 s, in both control (beige) and Talazoparib-treated cells (light blue) PARP1 is preferentially localized in high-density chromatin regions, likely due to the elevated local concentration of the Hoechst sensitizer (higher DNA density and higher concentration of AT-rich sequences) that concentrates the occurrence of DNA damage in these regions (Fig. 3c). As time progressed, control cells displayed a decreasing value of DNA density associated with PARP1, along with a decrease in colocalization with heterochromatin, consistent with PARP1 dynamic relocation into euchromatin or decreased chromatin binding. In contrast, Talazoparib-treated cells maintained PARP1 enrichment in high-density regions with sustained colocalization with heterochromatin, showing minimal redistribution over time (Fig. 3c). Consistently, analysis of DNA density and colocalization with heterochromatin in Fig. S1e reveals a comparable behavior when DNA damage is induced in a ROI inside the nucleus. As expected, in control cells, PARP1 shifts toward lower-density chromatin over time, whereas in Talazoparib-treated cells it remains confined to high-density, heterochromatic regions. Notably, this behavior mirrors that observed when DNA damage is induced at the nuclear periphery, indicating that PARP1 trapping upon PARP inhibition is largely independent of the nuclear sub-compartment and instead governed by the local chromatin context, thereby hindering PARP1 redistribution and downstream repair progression. Overall, these results highlight how chromatin context influences PARP1 dynamics and how PARP inhibitors interfere with its chromatin remodeling-dependent redistribution. Importantly, extending the analysis across multiple Talazoparib concentrations (Fig. S2) reveals a graded response, demonstrating that PARP1 redistribution and chromatin relaxation are not governed by a simple binary on/off mechanism but can be quantitatively captured across intermediate chromatin remodeling states.

### Hoechst-based confocal analysis captures chromatin remodeling dynamics in live cells

**Fig. 4:**
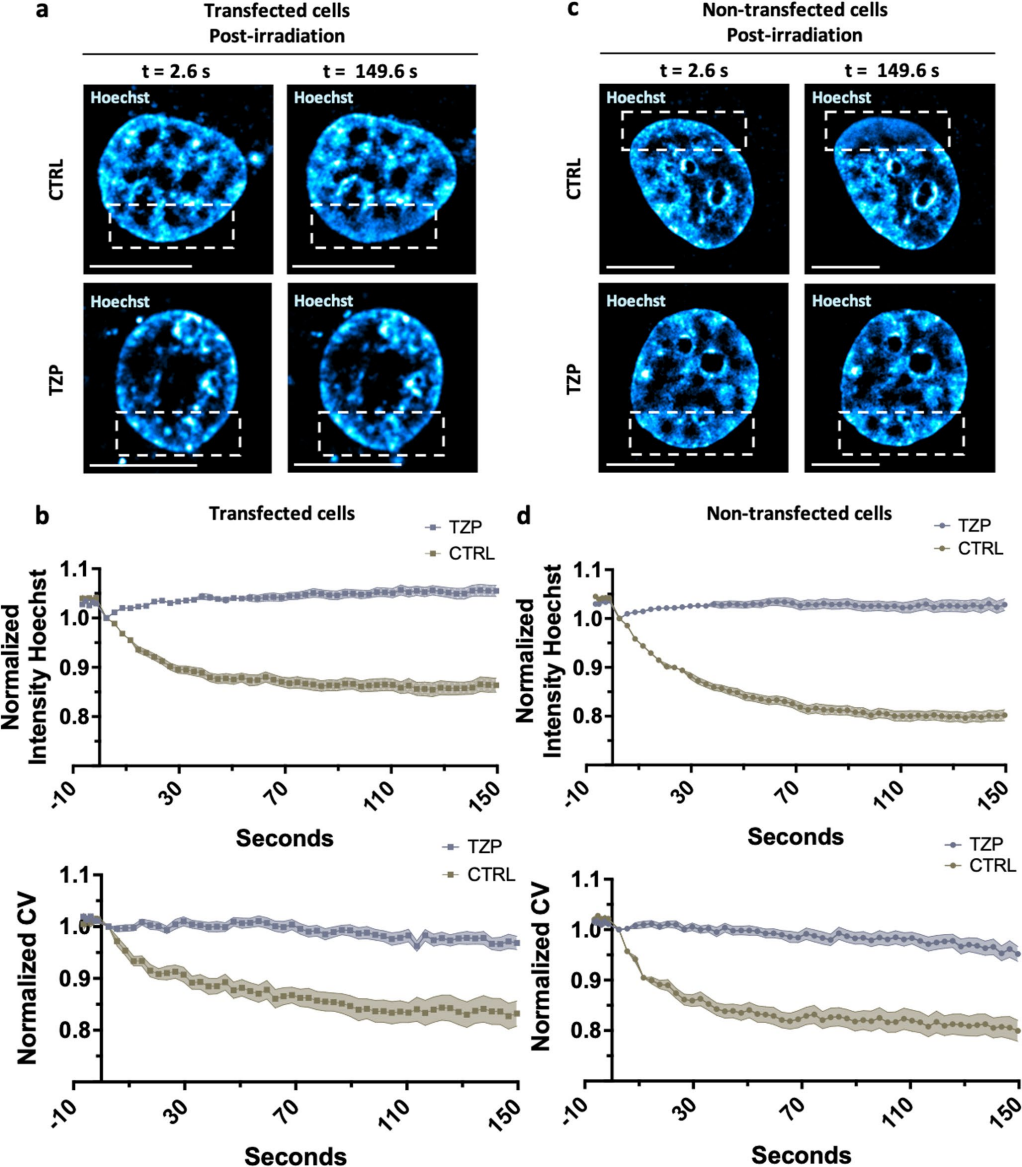
Single-channel imaging reveals chromatin relaxation after DNA damage and its inhibition by Talazoparib in both PARP1-transfected and non-transfected cells. (**a**) Representative confocal images of PARP1-transfected HeLa cells at post-irradiation time points (t = 2.6 s and 149.6 s) following laser-induced DNA damage (dashed square). Conditions include control (CTRL) and Talazoparib-treated cells (TZP). Staining: Hoechst (cyan-hot LUT) and PARP1 (not shown). Scale bars: 10 μm. (**b**) PARP1-transfected cells. Top: Quantitative analysis of normalized intensity of Hoechst in control (beige square) and Talazoparib-treated (gray square) cells. Pattern cloud plots show the mean ± s.e.m. of 3 independent experiments, with approximately 10 cells analyzed per experiment, for a total of ~ 30 cells. Bottom: Normalized coefficient of variation (CV) in control (beige square) and Talazoparib-treated (gray square) cells. Cloud plots show the mean ± s.e.m. of 3 independent experiments, with approximately 10 cells analyzed per experiment, for a total of ~ 30 cells. (**c**) Representative confocal images of non-transfected cells at post-irradiation time points (t = 2.6 s and 149.6 s) following laser-induced DNA damage (dashed square). Conditions include control and Talazoparib-treated cells. Staining: Hoechst (cyan-hot LUT). Scale bars: 10 μm. (**d**) Non-transfected cells. Top: Quantitative analysis of normalized intensity of Hoechst in control (beige circle) and Talazoparib-treated (gray circle) cells. Pattern cloud plots show the mean ± s.e.m. of 3 independent experiments, with approximately 10 cells analyzed per experiment, for a total of ~ 30 cells. Bottom: Normalized CV in control (beige circle) and Talazoparib-treated (gray circle) cells. Cloud plots show the mean ± s.e.m. of 3 independent experiments, with approximately 10 cells analyzed per experiment, for a total of ~ 30 cells

We next analyzed the Hoechst channel alone in both PARP1-transfected cells (Fig. 4a-b) and non-transfected cells (Fig. 4c-d). In PARP1-transfected cells (Fig. 4a), confocal imaging revealed a clear chromatin relaxation at the site of laser-induced DNA damage, visible as a localized reduction in Hoechst intensity that became progressively more homogeneous starting from the earliest post-irradiation time point (t = 2.6 s) until the final time point (t = 149.6 s). This relaxation pattern was absent in Talazoparib-treated cells, where chromatin remained visibly compacted in the damaged region and displayed a distinct pattern of euchromatic and heterochromatic subdomains.

Chromatin reorganization within the damaged region was quantitatively assessed using Hoechst-based metrics (Fig. 4b). Specifically, we monitored both the average Hoechst intensity and its coefficient of variation (CV), which together provide a readout of chromatin compaction. Higher values of these metrics correspond to more compact chromatin, whereas lower values indicate chromatin relaxation. As shown in Fig. 4b, values were normalized to the first post-irradiation time point. In control cells (beige squares), both Hoechst intensity and CV progressively decreased over time in the DNA damage region, consistent with dynamic chromatin relaxation. This trend was altered in Talazoparib-treated cells (grey squares), where Hoechst intensity remained stable and the CV showed only a minor reduction, suggesting that PARP inhibition restrains chromatin decompaction in response to DNA damage.

A similar response was observed in non-transfected cells (Fig. 4c-d). Quantitative analysis of Hoechst intensity and CV further confirmed these observations: in control cells (beige circle), both parameters decreased progressively, whereas in Talazoparib-treated cells (grey circle), they remained largely unchanged. All together, these observations demonstrate that even single-channel analysis of nuclear DNA staining provides informative and quantifiable insight into chromatin dynamics, offering a simple and minimally invasive readout to monitor structural changes in live cells. Notably, comparable results were obtained in both PARP1-transfected and non-transfected cells, underscoring the robustness and general applicability of this approach. A comparable analysis of the Hoechst channel alone is shown in Fig. S1f, where DNA density and heterogeneity were quantified within a DNA damage region located in the nuclear interior. In control cells (beige), Hoechst intensity and its coefficient of variation progressively decreased, consistent with chromatin relaxation, whereas in Talazoparib-treated cells (grey) both parameters remained largely stable, indicating restrained chromatin decompaction. This suggests that chromatin relaxation upon DNA damage is regulated independently of the intranuclear position of the lesion.

### Chromatin relaxation kinetics are comparable in transfected and non-transfected cells

**Fig. 5:**
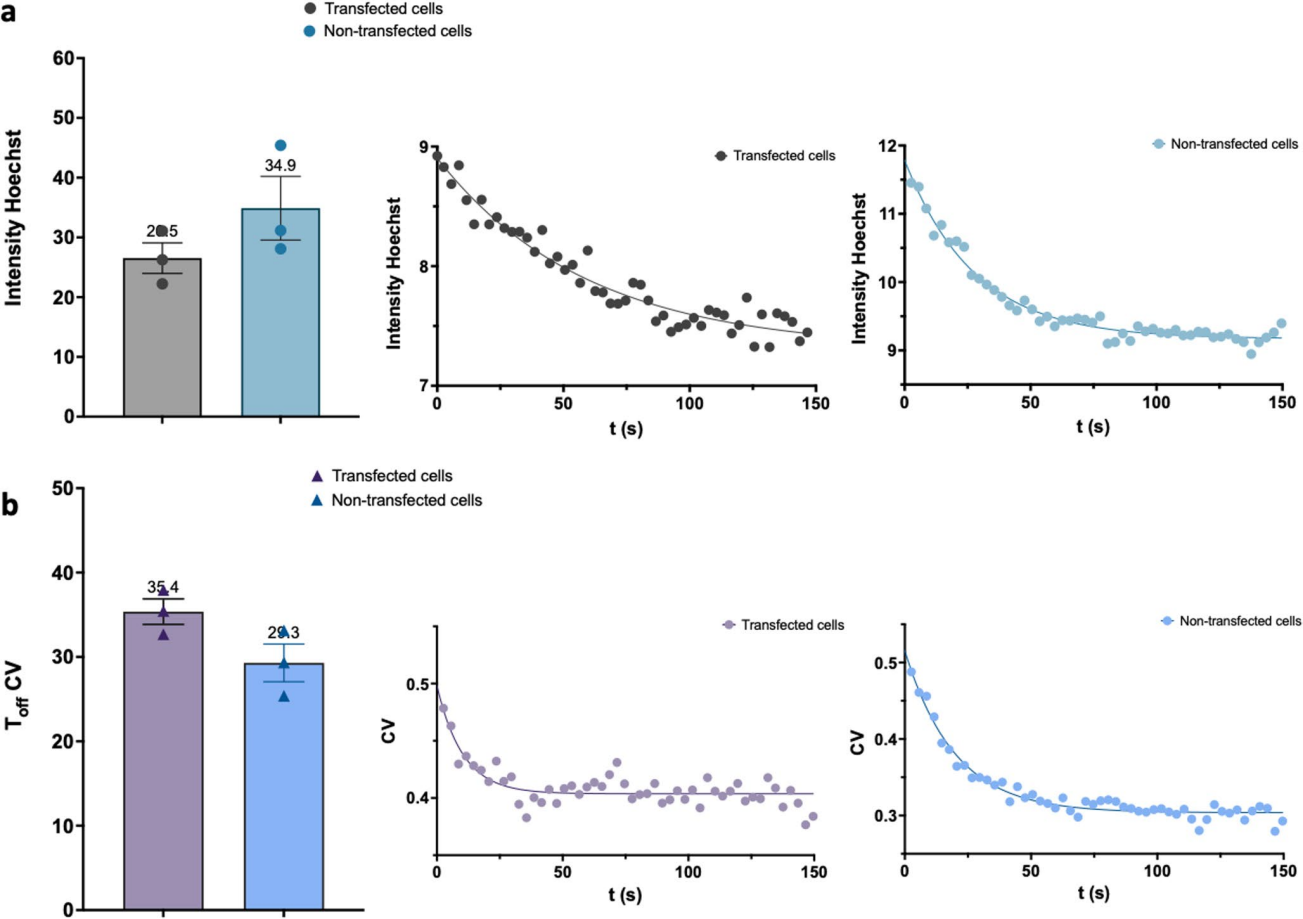
Comparable chromatin relaxation dynamics in PARP1-transfected and non-transfected cells. (**a**) Left: Dot plots showing the comparison of decay constants (T_off_) for Hoechst intensity in PARP1-transfected (grey circle) and non-transfected (light blue circle) cells. Data points represent the mean ± s.e.m. of 3 independent experiments, with approximately 10 cells analyzed per experiment. T-Test was applied and no significant difference was detected. Right: Representative decay fitting curves of Hoechst intensity over time in PARP1-transfected (grey circle) and non-transfected (light blue circle) cells. (**b**) Left: Dot plots showing the comparison of decay constants (T_off_) for Hoechst CV in PARP1-transfected (violet triangle) and non-transfected (blue triangle) cells. Data points represent the mean ± s.e.m. of 3 independent experiments, with approximately 10 cells analyzed per experiment. T-Test was applied and no significant difference were detected. Right: Representative decay fitting curves of Hoechst CV over time in PARP1-transfected (violet circle) and non-transfected (blue circle) cells

Finally, we quantified the decay kinetics of Hoechst intensity and CV over time in both control PARP1-transfected (Fig. 5a) and non-transfected cells (Fig. 5b). Specifically, we calculated the T_off_ values (exponential decay constants) to quantify the kinetics of chromatin relaxation and to directly compare the temporal dynamics between PARP1-transfected and non-transfected cells. As shown in Fig. 5a, the average T_off_ for Hoechst intensity was 26.52 s in PARP1-transfected cells (grey) and 34.88 s in non-transfected cells (light blue), as illustrated by the representative fits. The difference was not statistically significant, indicating that the kinetics of Hoechst signal decay, reflecting chromatin decompaction, were comparable between the two conditions. Similarly, in Fig. 5b, the T_off_ values for the coefficient of variation were 35.36 s in PARP1-transfected cells (violet) and 29.29 s in non-transfected cells (blue). These comparable values suggest that the dynamics of chromatin heterogeneity within the damaged region evolve at a similar pace in both cell populations. Importantly, this validation confirms that informative measurements can be obtained using the Hoechst channel alone, without the need for PARP1-RFP transfection, thereby simplifying the workflow and extending the applicability of this approach. These data also confirm that expression of the PARP1 chromobody [2] does not interfere with the chromatin relaxation process that follows DNA damage induction.

### Discussion/Conclusions

DNA damage elicits a well-characterized cascade of chromatin remodeling events that promote lesion accessibility, coordinate the recruitment of repair factors, and ensure genome stability [3]. These early chromatin changes, most notably the relaxation of chromatin at the damage site, are a hallmark of the DNA damage response (DDR). However, it remains unclear whether, and to what extent, such remodeling events occur uniformly [35].

To study these dynamics in live cells, we set up a relatively simple approach based on confocal microscopy and staining with a Hoechst dye used both as a sensitizer for laser micro-irradiation and a marker of chromatin remodeling. By carefully optimizing dye concentration and illumination parameters, we defined a protocol to induce DNA damage with minimal photobleaching of the Hoechst dye, enabling reliable live-cell monitoring of chromatin remodeling via changes in Hoechst fluorescence intensity and coefficient of variation (CV).

To investigate the pattern of laser-induced DNA damage, we applied a time-resolved version of our QUANDO method, recently published by Paternò et al. [27]. QUANDO previously showed that in U937-PR9 cells, spontaneous and PML-RARα–induced damage localizes mainly to euchromatin, while Neocarzinostatin-induced damage is more evenly distributed [27]. Here, we found that PARP1 initially accumulated in more compact chromatin domains and later redistributed to euchromatic regions in control cells, while remaining confined to dense chromatin regions under Talazoparib. Control cells showed progressive chromatin relaxation at damage sites, indicated by decreased Hoechst intensity and coefficient of variation (CV), whereas Talazoparib blocked this relaxation. Similar chromatin dynamics in PARP1-transfected and non-transfected cells confirmed that Hoechst staining alone effectively monitors chromatin remodeling. Overall, chromatin remodeling occurs dynamically after DNA damage but is hindered by PARP inhibition, and Hoechst analysis offers a minimally invasive live-cell method to track these changes.

Previous studies have investigated chromatin remodeling during DNA damage response using various microscopy-based approaches. For instance, Izhar et al. [15] reported, similarly to our observations, that UV laser micro-irradiation combined with Hoechst staining induces localized chromatin relaxation (called “antistripes” by the authors) mediated by PARP1. However, their analysis was qualitative and did not provide spatial and temporal quantification [15]. Several studies have exploited labeling of histones with photoconvertible fluorescent proteins and time-lapse imaging [3, 18, 34]. Fluorescence correlation spectroscopy (FCS) has been used to quantify the mobility of inert or chromatin-associated proteins within the chromatin environment [12, 21, 23]. Förster Resonance Energy Transfer (FRET) imaging has been used to measure chromatin compaction at the nanoscale following induction of DNA damage [22, 28].

In this framework, our method provides a simpler, yet quantitative, way to analyze chromatin remodeling in live cells during the early DNA damage response. By labeling chromatin with a DNA dye, it avoids the need for photoactivatable fluorescent histones. However, this also constitutes a limitation, as chromatin states are defined based only on DNA density, without incorporating epigenetic markers. In this respect, future integration of epigenetic markers could offer deeper insights into the relationship between chromatin states and DNA damage dynamics. Our protocol requires a region of interest (ROI) with a size of at least a few microns (the smallest ROI that we have tested has an area ~ 24 μm²), whereas studies using fluorescent histones have reported ROIs smaller than ~ 10 μm². Despite this constraint, our approach demonstrates sufficient sensitivity to capture gradual changes in chromatin relaxation dynamics in response to different Talazoparib concentrations.

Looking ahead, this approach could be combined with live-cell super-resolution imaging techniques, such as image scanning microscopy (ISM) or Airyscan microscopy, to enhance spatial resolution while preserving live-cell compatibility and minimizing phototoxicity [4, 9, 16]. Its versatility also makes it suitable for application in more physiologically relevant systems, including 3D spheroids that better recapitulate tissue architecture and nuclear organization. Overall, this work opens new avenues to dissect the interplay between chromatin architecture and DNA repair dynamics with minimal experimental perturbation.

## Electronic Supplementary Material

Below is the link to the electronic supplementary material.

Supplementary Material 1

## Data Availability

Data sets generated during the current study are available from the corresponding author on reasonable request.
