# Supplementary material for: Tracking DNA damage localization and chromatin remodeling in live cells using time-resolved quantitative analysis of DNA counterstains
Source: Cell Mol Life Sci. 2026 Feb 16;83(1):115. doi: 10.1007/s00018-026-06100-9 (PMC12913851; doi:10.1007/s00018-026-06100-9)
Supplement: Supplementary file 1 — Supplementary Material 1 [file 18_2026_6100_MOESM1_ESM.docx]

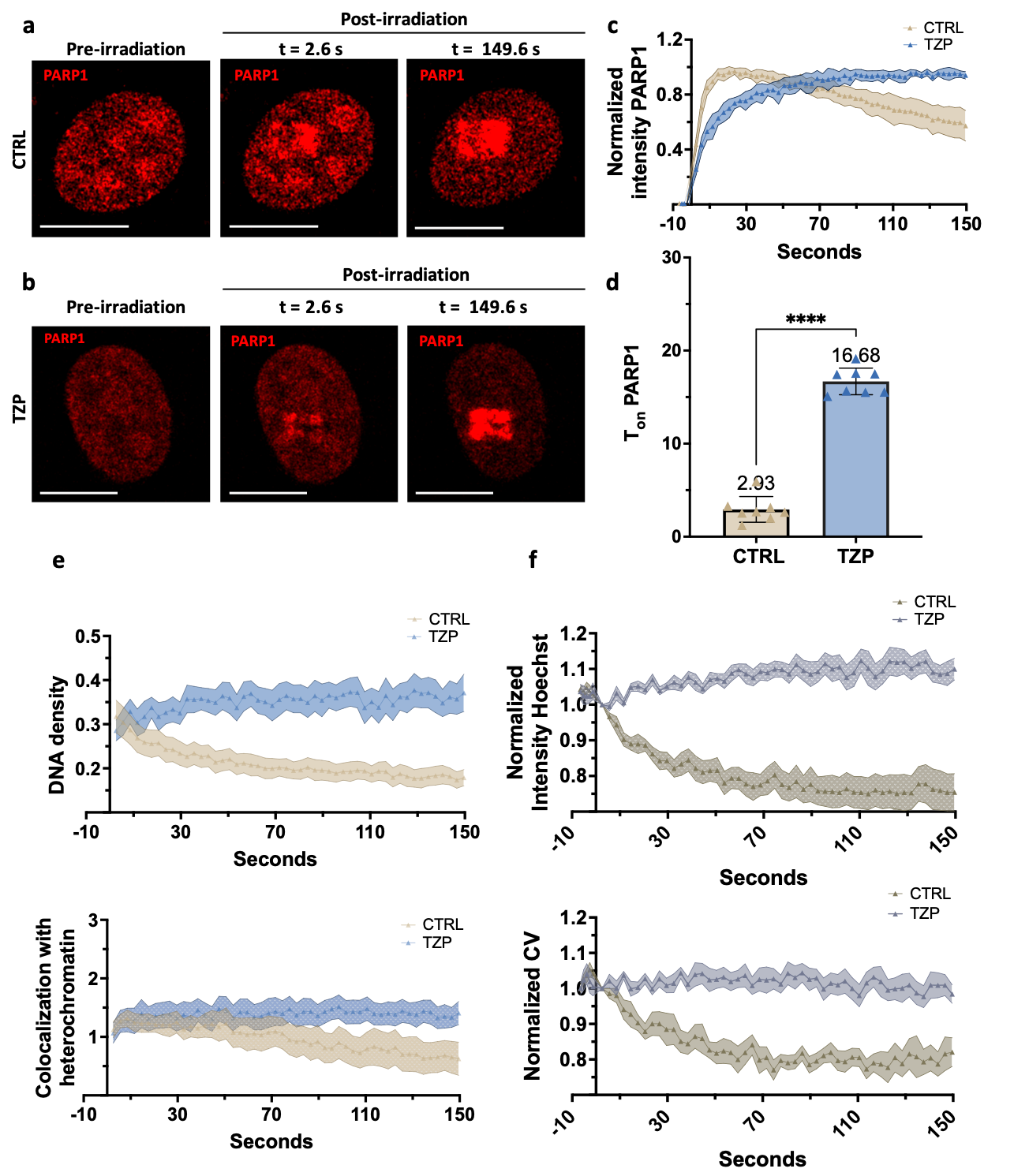


**Figure S1.** **Intranuclear laser-induced DNA damage reveals PARP1 recruitment and chromatin dynamics.**
(a-b) Representative confocal images of HeLa cell nuclei showing PARP1 (red) distribution before and after intranuclear laser-induced DNA damage in control and Talazoparib-treated cells. For each condition, images show PARP1 localization at pre-irradiation and post-irradiation time points within a defined intranuclear region of interest. Scale bars: 10 µm. c) Normalized PARP1 intensity profiles over time in control and talazoparib-treated cells. Cloud plots show the mean ± s.d. from 8 cells. d) Dot plots showing the comparison of PARP1 Tₒₙ values between control and Talazoparib-treated cells. Data points represent mean ± s.d. from 8 cells, T-Test **** p<0.0001. (e) Top: DNA density corresponding to PARP1 accumulation over time in control (beige) and Talazoparib-treated (blue) cells. Cloud plots show the mean ± s.e.m. of 8 cells. Bottom: Colocalization of PARP1 with heterochromatin over time in control (beige) and Talazoparib-treated (blue) cells. Pattern cloud plots show the mean ± s.e.m. of 8 cells. **(f) Top: Quantitative analysis of normalized intensity of Hoechst in control (beige) and Talazoparib-treated (gray) cells. Pattern cloud plots show the mean ± s.e.m. of** 8 cells. **Bottom: Normalized coefficient of variation (CV) in control (beige) and Talazoparib-treated (gray) cells. Cloud plots show the mean ± s.e.m. of** 8 cells.


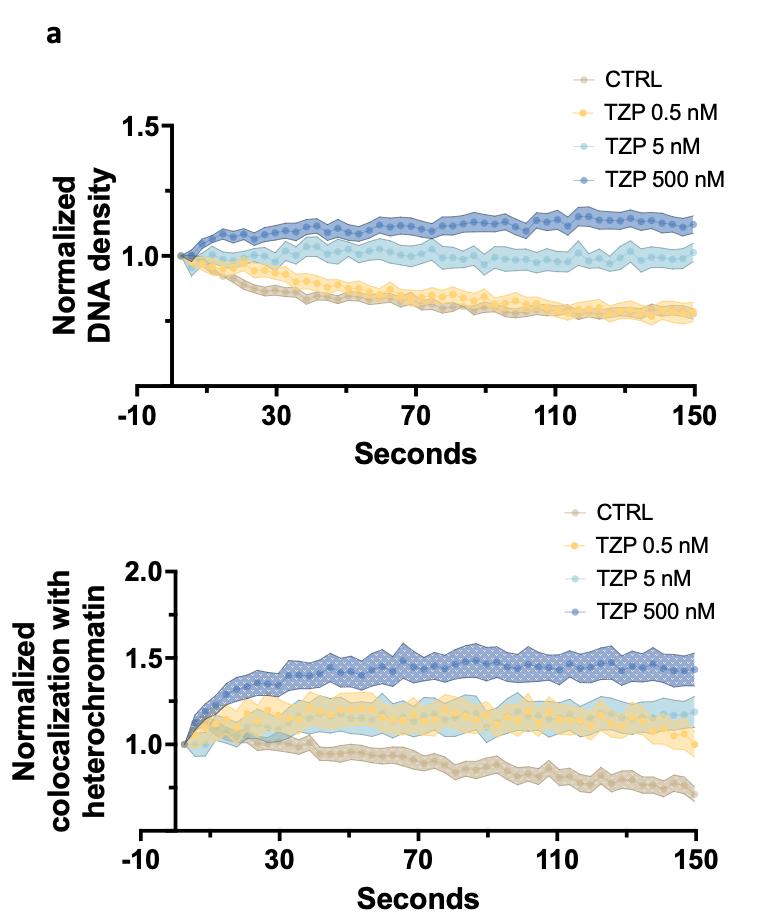


**Figure S2. PARP1 localization dynamics revealed by time-resolved QUANDO in response to different Talazoparib concentrations.** (a) Top: Normalized DNA density associated with PARP1 accumulation over time in control (beige), 0.5 nM (yellow), 5 nM (light blue), and 500 nM (blue) talazoparib-treated cells. Cloud plots show the mean ± s.e.m. from 8 cells. Bottom: Normalized PARP1 colocalization with heterochromatin over time under the same conditions. Pattern cloud plots show the mean ± s.e.m. from 8 cells.
